# Breaking barriers? Ethnicity and socioeconomic background impact on early career progression in the fields of ecology and evolution

**DOI:** 10.1002/ece3.6423

**Published:** 2020-06-08

**Authors:** Klara M. Wanelik, Joanne S. Griffin, Megan L. Head, Fiona C. Ingleby, Zenobia Lewis

**Affiliations:** ^1^ Department of Evolution, Ecology, and Behaviour School of Life Sciences University of Liverpool Liverpool UK; ^2^ Research School of Biology College of Science The Australian National University Canberra ACT Australia

**Keywords:** career progression, early career researchers, ethnic minorities, intersectionality, socioeconomic background, women in science

## Abstract

The academic disciplines of Science, Technology, Engineering and Mathematics (STEM) have long suffered from a lack of diversity. While in recent years there has been some progress in addressing the underrepresentation of women in STEM subjects, other characteristics that have the potential to impact on equality of opportunity have received less attention. In this study, we surveyed 188 early career scientists (ECRs), defined as within 10 years of completing their PhD, in the fields of ecology, evolutionary biology, behaviour, and related disciplines. We examined associations between ethnicity, age, sexual orientation, sex, socioeconomic background, and disability, with measures of career progression, namely publication record, number of applications made before obtaining a postdoc, type of contract, and number of grant applications made. We also queried respondents on perceived barriers to progression and potential ways of overcoming them. Our key finding was that socioeconomic background and ethnicity were associated with measures of career progression. While there was no difference in the number of reported first‐authored papers on PhD completion, ethnic minority respondents reported fewer other‐authored papers. In addition, ECRs from a lower socioeconomic background were more likely to report being in teaching and research positions, rather than research‐only positions, the latter being perceived as more prestigious by some institutions. We discuss our findings in the context of possible inequality of opportunity. We hope that this study will stimulate wider discussion and help to inform strategies to address the underrepresentation of minority groups in the fields of ecology and evolution, and STEM subjects more widely.

## Graphical abstract

This study provides evidence that socioeconomic background and ethnicity can impact negatively on the career progression in early career researchers in the field of ecology and evolution.

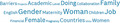

## 1 INTRODUCTION

Diversity in the workplace can have a positive impact on the output of the workforce. In the corporate and social sciences sectors, numerous studies have shown that a more balanced workforce, in terms of gender and ethnicity, performs better, in terms of outputs, growth, and financial gains (e.g., Herring, 2009; Herring, 2017; Hunt et al., 2018; Rohner & Dougan, 2012). To date, similar impacts of diversity in Science, Technology, Engineering and Mathematics (STEM) academia have been less well studied, which represents a significant gap in the literature (Valantine & Collins, 2015). However, studies suggest that higher departmental diversity is related to higher placing in institutional rankings (Herring, 2014), gender‐diverse collaborative groups produce higher quality science (Campbell et al., 2013), and ethnically diverse groups produce papers with higher scientific impact (AlShebli, Rahwan, & Woon, 2018).

The disciplines of STEM have historically suffered from an underrepresentation of marginalized groups, defined in the UK as protected groups under the 2010 UK Equality Act. According to the 2015–2016 UK Higher Education Statistics Agency (HESA) data, academics working in STEM subjects were 41.4% female, while 51% of the national population is women. Where the data are broken down according to role, women are represented much less than men in senior positions, even though at undergraduate level female students outnumber male students; this loss of female representation with academic progression has been dubbed the “leaky pipeline” effect (Pell, 1996; Sugimoto et al., 2013). Internationally, only 32% of researchers in STEM are female in Western Europe and North America, and 29% worldwide (UNESCO Institute for Statistics, 2017). With respect to other minority groups, the figures are even more stark. According to 2015–2016 UK HESA statistics, STEM academics were 10.3% nonwhite, and 0.03% disabled, in contrast to the student demographics for the same time period, of 21% and 11%, respectively; in 2016–2017, only 0.6% of UK professors were black compared to 3.3% of the overall population.

The underrepresentation of minority groups is the result of a multitude of complex, often multifaceted, barriers. For example, individuals from minority groups are more likely to have negative perceptions of their own academic career success (Paul, 2016), less likely to obtain research funding (reviewed in Vasquez et al., 2006), and have lower likelihood of being promoted (e.g., Bhatt, 2013). The fact that childcare and caring responsibilities still overwhelmingly lie with women, coupled with the fact that HE institutions are not always “family‐friendly,” is likely to be a major barrier for many female academics (e.g., Gaio Santos, 2016; King, 2008). Underrepresented groups may be less able to access voluntary positions and internships to gain experience and training due to financial insecurity or reduced social capital (reviewed in Fournier & Bond, 2015) and are less likely to have role models (Hermann et al., 2016). Even if individuals from minority groups obtain a permanent academic position, they are more likely to report being unsatisfied with their career, and to leave the sector (Palepu et al., 2000).

Single‐category analyses that solely focus on ethnicity, gender, or disability, for example, overlook individuals who identify with more than one protected characteristic. The challenges faced by individuals can be further compounded, as described by intersectional theory (Armstrong & Jovanovic, 2017; Crenshaw, 1989; Malcolm et al., 1976; Ong et al., 2011). The concept of intersectionality is rooted in Black feminist culture and arose during the 1960s in response to deep racial, gender, and class divides (The Combahee River Collective, 2001). Later, intersectionality emerged in the academic literature (Crenshaw, 1989). The term intersectionality avoids isolating identities into mutually exclusive structures of inequality and is now used broadly across academic disciplines. Intersectional research suggests that ethnic minority female academics are more likely to suffer from self‐doubt and are more likely to experience challenges to their authority, compared to white female academics. This is in part due to the fact that they suffer discrimination against both their sex and their ethnicity, demonstrating the importance of considering protected characteristics together, rather than in isolation. However, intersectional theory goes further than this, arguing that an individual's identities can also interact, resulting in an experience which is “greater than the sum of its parts” (Crenshaw, 1989).

In the present study, we apply intersectional principles to the study of academic institutions. We present data collected from a survey with 188 respondents, all early career researchers (ECRs) in the fields of ecology, evolutionary biology, behavior, and related disciplines (the fields of the authors). Although the definition of ECR varies across countries and organizations, we used a reasonably common variant, defined as individuals within 10 years of completing their PhD. We examined associations between six characteristics—ethnicity, age, sexual orientation, sex, socioeconomic background, and disability—and four measures of career progression, publication record, number of applications made before obtaining a postdoc, type of contract, and number of grant applications made. We included the interactions between these factors, where possible, to explore intersectionality. Socioeconomic background is not currently a characteristic protected by the UK Equality Act 2010; therefore, for the remainder of the paper, we refer to the characteristics we examined as “characteristics of interest.” We included socioeconomic background in our study as it has been suggested that financial barriers can make it harder for early career researchers to progress in science (National Academy of Sciences, National Academy of Engineering, & Institute of Medicine, 2011). We asked whether respondents came from a lower socioeconomic background, presenting them with the legal UK definition of the term (Office for National Statistics, 2010). We also queried respondents on perceived barriers to career progression, and report practical suggestions, based on their experiences, for overcoming said barriers. We pitched our study in the context of academic research in the fields of ecology, evolutionary biology, behavior, and related disciplines and therefore examine career progression within this context.

## 2 METHODS

To obtain information on the barriers faced by early career scientists in the fields of ecology, evolutionary biology, behavior, and related disciplines, we conducted an online survey (questions given in Table 1), hosted by SurveyMonkey, Inc. (USA). The link to the survey was communicated via social media and email, via Evolutionary Directory (EvolDir), with a simple title “STEM survey.” We left the survey open for 3 weeks during which time we received 188 anonymous responses. Ethical approval to collect the responses was required and granted by the University of Liverpool (application reference number 2229). The responses were collected anonymously and voluntarily. We specified that respondents should be early career scientists within a maximum of 10 years of completing their PhD. For transparency, a set of questions was outlined in a research plan prior to analysis. This plan also included rules for data cleaning and detailed methods for answering each of these questions, which are described below (original research plan available at: https://github.com/kwanelik/Breaking‐barriers).

**TABLE 1:** Table of survey questions (and format of answers)

**Survey questions** How old are you? (multiple choice) What is your gender? (multiple choice) What is your sexual orientation? (multiple choice) What is your nationality? (multiple choice) What is your ethnic background? (multiple choice) Do you consider yourself to have originally come from a lower socio‐economic background, as defined by the National Statistics Socio‐economic Classification 2005 (or similar if you are non‐UK based)? See here for details: https://www.ons.gov.uk/methodology/classificationsandstandards/otherclassifications/thenationalstatisticssocioeconomicclassificationnssecrebasedonsoc2010.^a^ (multiple choice) Are you deemed to have a disability, as defined under the Disability Discrimination Act 2010 (or similar if you are non‐UK based)? See here for details: https://www.gov.uk/definition‐of‐disability‐under‐equality‐act‐2010. (multiple choice) From what country were you awarded your PhD? (multiple choice) In which year did you hand in your PhD thesis? (multiple choice) How old were you when you handed in your PhD thesis? (multiple choice) How many first‐author peer reviewed publications did you have accepted when you handed in your PhD? (free text) How many other‐author peer reviewed publications did you have accepted when you handed in your PhD? (free text) How many postdoc applications did you make before (and including) your first postdoc job (if applicable)? (free text) How many postdocs have you done, including current one (if applicable)? (free text) What type of contract are you currently employed on? (multiple choice) Are you on a permanent academic contract? (multiple choice) If yes, how many applications for permanent positions did you make before (and including) obtaining your current position? (free text) If yes, how many years post‐PhD were you when you obtained your current position? (free text) How many grant applications (incorporating a salary for yourself) have you made in total? (free text) In the past/currently have you faced/are there any barriers with regards to your identity during the course of your career? (multiple choice) If so, what do you feel was the most important barrier? (free text) If you were able to overcome the barrier stated in the previous question, how did you do this? (free text) Do you have any other comments pertinent to this study? (free text)

^a^ We included this link to help respondents decide whether or not they classified themselves as coming from a lower socioeconomic background. We did not include the full set of questions needed to derive the National Statistics Socio‐economic classification (NS‐SEC) in our survey.

### 2.1 Summary of respondent demographics

Respondents to our survey were geographically diverse. They included respondents who had completed their PhD in the United States and Canada (*n* = 64), Europe (*n* = 55), Australia and New Zealand (*n* = 20), or Central/South America (*n* = 5). The modal age of respondents was 30–34 and the modal age upon PhD submission was 25–29. In terms of types of contracts, research‐only contracts were the most represented (*n* = 76; as opposed to teaching‐only or research and teaching combined contracts) and were not on a permanent contract at the time of completing the survey (*n* = 107). Approximately equal numbers of respondents reported either having faced barriers (*n* = 71) or not (*n* = 60). A full breakdown of numbers of respondents in relation to these characteristics of interest is provided in Table S1. Note that not all respondents answered all questions.

### 2.2 Data cleaning

In some cases, answers given by respondents were ambiguous. Where respondents included a lower limit (e.g., number of postdoc applications made before being awarded a position = “100+”), this was used. Where they included a range (e.g., “15–20”), a mean value was used. Where they included an approximate figure (e.g., “approx. 100”), this was treated the same as an exact figure. Where decimal numbers were given (e.g., “0.5”), these were rounded up. Sixteen responses were excluded due to ambiguous answers which could not be confidently interpreted. As detailed in our research plan (see above), outliers were defined as those data points lying more than three standard deviations from the mean and were removed prior to each analysis. The number of outliers removed varied between analyses but was consistently low (*n* = 0–4). Some groups with very low representation, for example, “other/prefer not to answer” responses, also had to be removed due to problems with model convergence. Individuals with missing values for any independent variables of interest were also excluded. Final sample sizes for each analysis are summarized in Table S2 and detailed below.

### 2.3 Statistical modeling

Generalized linear models (GLMs) were used to look for associations between six characteristics of interest: ethnicity, age, sexual orientation, sex, socioeconomic background, and disability, and five measures of career progression.

#### 2.3.1 Publication record

We looked for associations between the characteristics and the number of first‐ and other‐author papers published upon PhD submission (sample size: *n* = 144). Journal of publication was not considered, and we did not account for variation in the length of PhD. The number of first‐ and other‐author papers upon PhD submission were modeled separately. Both were overdispersed. A better fit was therefore achieved using a GLM with a negative binomial error distribution (Table S2).

#### 2.3.2 Number of applications made before obtaining a postdoc

We looked for associations between the characteristics and the number of applications made before commencing an advertised postdoc position or fellowship (combined; *n* = 126). Because we were interested in relating success to effort, we excluded those respondents who gave the number of applications, but later stated that they had not yet been successful in securing a postdoc (*n* = 3). The number of applications made before obtaining a postdoc was overdispersed so we used a GLM with a negative binomial error distribution (Table S2).

#### 2.3.3 Types of contract

We looked for associations between the characteristics and the type of contracts respondents were on (either research‐only or teaching and research; *n* = 111). As only 5 respondents were on teaching‐only contracts, these were excluded from the analysis. We used a GLM with a binomial error distribution (Table S2). We also looked for associations between these characteristics and whether an individual was on a permanent or temporary contract (*n* = 139). Again, we used a GLM with a binomial error distribution (Table S2).

#### 2.3.4 Number of grant applications made

We looked for associations between the characteristics and the number of grant applications made (*n* = 122). We excluded small grants (e.g., travel grants), by specifically asking respondents about grants which included their salary. The number of grant applications was overdispersed, so we used a GLM with a negative binomial error distribution (Table S2).

#### 2.3.5 Reported barriers

We looked for associations between the characteristics and whether or not an individual reported facing barriers to their identity (*n* = 133). We used a GLM with a binomial error distribution (Table S2).

All analyses were conducted in R version 3.4.4 (R Core Team, 2018). All models included the six characteristics as fixed effects (ethnicity, age, sexual orientation, sex, socioeconomic background, and disability). Due to limited sample sizes, only interactions between sex and the other characteristics were included (where possible; see Table S2). We collected data on the country of PhD, with an aim to account for any geographical variation in our analyses. However, we did not include this variable in our final analyses, primarily to avoid overfitting (models including country as a random effect did not converge). Year of PhD completion was included to account for any temporal autocorrelation. We originally planned for this variable to be included as a random effect but mixed effect models did not converge (likely because of the limited sample size). Therefore, years were combined into five bins of approximately equal size (2007–2009, 2010–2011, 2012–2013, 2014–15, 2016–2017) and included as a fixed effect. Other additional fixed effects included total publications at the time of PhD submission, total number of postdocs completed, whether or not on a permanent contract and interactions between these variables and sex.

Some adjustments to model specifications had to be made due to problems with model convergence. No interaction terms could be included in the analysis of reported barriers (see Table S2). Age was also binned into two main groups (<34 and >34 years) in the analysis of type of contract (permanent or temporary). All fixed effects within a model were checked for collinearity by computing generalized variance inflation factors (GVIFs; Fox & Monette, 1992). Fixed effects excluded on these grounds were year of PhD completion (type of contracts—research‐only or teaching and research) and age (type of contracts—research‐only or teaching and research, number of grant applications made and reported barriers; Table S2). All models were checked for normal and homoscedastic residuals.

Sets of candidate models were generated from each global model, which included all of the fixed and interaction terms of interest (Table S2) using the MuMIn package (Bartoń, 2014). All candidate models were then ranked on relative fit using the Akaike information criteria corrected for small sample sizes, AICc (Hurvich & Tsai, 1989). Those with a ΔAICc < 2 relative to the lowest value were considered to be equally supported as the best models to explain the data (top models, S3). Effect sizes, unconditional standard errors and estimated *p*‐values were obtained by averaging across this set of top models using the zero method (Burnham & Anderson, 2002). Model averaging was carried out because multiple models, equally supported as the best models to explain the data, were found rather than a single best model (Table S3). All reported effect sizes are on a transformed scale. Where two or more numeric variables were present in an averaged model, these were standardized using two *SD* (Gelman, 2008) to make them directly comparable. The relative importance of a variable was taken to be the sum of the Akaike weights of the top models in which it was found (R Core Team, 2018). Variables that appear in one or more top model, but are not significant, are still reported. Even though there is no evidence for such variables affecting the response, they are still considered useful in predicting point estimates (Grueber et al., 2011).

### 2.4 Word frequency analysis

All free text answers from respondents on (a) the most important barriers they have faced, and (b) how they overcame these barriers were analyzed using the text mining (tm) package (Feinerer & Hornik, 2018; Feinerer et al., 2008) in R version 3.4.4 (R Core Team, 2018). Briefly, text was transformed and cleaned (removing all numbers, punctuation, and stopwords). Then, text‐stemming was performed and frequencies of root words were calculated. The most frequently used words are reported. As these were related to one of the characteristics of interest (see Results), we then tested for an association between that characteristic and the use of the words using a chi‐squared test.

## 3 RESULTS

### 3.1 What do the numbers suggest with regard to barriers to career progression faced by ECRs?

#### 3.1.1 Publication record

There were no significant associations between the characteristics of interest and the reported number of first‐author papers published upon submission of PhD. However, there was a significant association between the number of other‐author papers reported to have been published upon PhD submission and ethnicity, with ethnicity appearing in all four top models (Table S3). Both Black, Asian and minority ethnic (BAME; *p* < .01) and Hispanic‐Latino individuals (*p* = .04) reported finishing their PhD with approximately one less other‐author publication than individuals of white ethnic background (Table 2). Given that the majority of respondents to our survey were awarded their PhDs in geographical regions where the most frequent ethnic group is White, these results suggest that coming from less frequent (minor) ethnic groups is associated with having fewer other‐author publications.

**TABLE 2:** Model‐averaged, transformed parameter estimates, unconditional standard errors, estimated *p*‐values and relative importance of predictors of (i) number of first‐author papers on PhD submission, (ii) number of other‐author papers on PhD submission, (iii) number of applications made before obtaining a postdoc, (iv) research versus teaching and research contract, (v) permanent contract or not, (vi) number of grant applications made, and (vii) reported barrier or not

Response	Parameter^a^	Model‐averaged estimate^b^	Unconditional *SE*	Estimated *p*‐value	Relative importance
Publication record					
No. of first‐author papers	(Intercept)	1.05	0.14	<.001	‐
	Ethnic group Latino	−0.14	0.28	.62	0.30
	Ethnic group Other	0.15	0.30	.63	”
	Ethnic group White	−0.02	0.13	.86	”
	Socio Prefer not answer	0.08	0.34	.81	0.43
	Socio Yes	−0.11	0.16	.48	”
	Disability Yes	0.07	0.18	.70	0.26
No. of other‐author papers	(Intercept)	0.63	0.09	<.001	‐
	**Ethnic group BAME**	**−1.10**	**0.42**	**<.01**	**1.00**
	**Ethnic group Latino**	**−0.73**	**0.36**	**.04**	”
	Ethnic group Other	0.57	0.40	.16	”
	Sex Male	0.05	0.11	.68	0.28
	Disability Yes	−0.08	0.23	.73	0.24
No. of application before obtaining a postdoc	(Intercept)	1.67	0.28	<.001	‐
	**Total publications PhD**	**−0.07**	**0.03**	**.02**	**1.00**
	Sex Male	0.30	0.50	.55	0.59
	Socio Yes	0.04	0.12	.76	0.21
	Age PhD 25–29	0.05	0.21	.82	0.11
	Age PhD 30–34	0.07	0.25	.80	”
	Age PhD 35–39	−0.09	0.34	.80	”
	Age PhD 40–44	−0.15	0.57	.79	”
	Age PhD 25–29 × Sex Male	−0.09	0.37	.80	0.11
	Age PhD 30–34 × Sex Male	−0.19	0.60	.76	”
	Age PhD 35–39 × Sex Male	0.00	0.00	‐	”
	Age PhD 40–44 × Sex Male	0.00	0.00	‐	”
	Disability Yes	0.06	0.24	.79	0.19
	Sex Male × Total pub	0.00	0.02	.86	0.09
Types of contract					
Research versus Teaching and research	(Intercept)	−3.48	1.60	.03	‐
	Disability Yes	−1.49	0.00	1.00	0.88
	Sexual orientation Straight	1.07	1.46	.47	0.51
	**Permanent Yes**	**5.17**	**1.06**	**<.001**	**1.00**
	Sex Male	−0.67	0.76	.38	0.88
	**Socio Yes**	**1.61**	**0.72**	**.03**	**1.00**
	Disability Yes × Sex Male	0.33	0.00	.99	0.88
	Total postdocs	0.13	0.29	.65	0.30
	Total publications PhD	0.01	0.04	.84	0.11
Permanent or temporary contract	(Intercept)	−0.36	0.34	.29	‐
	**Age current < 34**	**−1.55**	**0.37**	**<.001**	1.00
	Total postdocs	−0.79	0.43	.06	1.00
	**Total publications PhD**	**1.19**	**0.32**	**<.01**	1.00
	Sex Male	−0.12	0.29	.68	0.39
	Total postdocs × Sex Male	−0.39	0.80	.63	0.25
	Disability Yes	0.12	0.44	.78	0.14
	Sexual orientation Other	−0.05	0.27	.84	0.14
	Sexual orientation Straight	−0.07	0.31	.83	”
No. of grant applications made	(Intercept)	1.72	0.29	<.001	‐
	Socio Yes	0.19	0.24	.43	0.55
	Years 2010–2011	−0.28	0.32	.38	1.00
	Years 2012–2013	−0.44	0.33	.18	”
	**Years 2014–2015**	**−1.07**	**0.30**	**<.001**	”
	**Years 2016–2017**	**−0.76**	**0.30**	**.01**	”
	Sex Male	−0.07	0.16	.66	0.28
	Sexual orientation Straight	0.06	0.20	.75	0.21
	Disability Yes	−0.03	0.16	.87	0.09
Reported barriers					
Reported barrier or not	(Intercept)	4.89	1.40	<.001	‐
	**Sexual orientation Other**	**−4.13**	**1.58**	**.01**	**1.00**
	**Sexual orientation Straight**	**−3.91**	**1.19**	**<.01**	”
	**Sex Male**	**−2.30**	**0.55**	**<.001**	**1.00**
	**Socio Yes**	**1.93**	**0.60**	**<.01**	**1.00**
	Years 2010–2011	−0.64	0.81	.43	0.78
	Years 2012–2013	−0.05	0.72	.95	”
	Years 2014–2015	−1.23	0.90	.17	”
	Years 2016–2017	−1.39	0.98	.16	”
	Disability Yes	0.18	0.71	.81	0.21

Note

Significant effects shown in bold.

^a^ Sexual orientation LGBT+, Sex Female, Socio No, Disability No, Age current >34, Permanent No, Age PhD 18–24, Year PhD 2007–2009, Total publications = 0 and Total postdocs = 0 (except for “Types of contracts” where mean values for Total publications and Total postdocs) were the reference categories.

^b^ Model‐averaged estimates are transformed, and for “Types of contracts” standardized using two *SD* (Hurvich & Tsai, 1989) for numeric variables (see Methods for details).

However, not all of the respondents to our survey were awarded their PhDs in such regions. Furthermore, certain ethnicities were more likely to have undertaken their PhD in certain regions (Fisher's exact test; *p* = .02; Figure S1) where expected outcomes from a PhD may differ. Therefore, in order to confirm this result and to try to disentangle ethnicity from geographical region, we ran an additional analysis on a subset of our data, only including individuals from regions where the most frequent ethnic group is White. In this analysis, we replaced ethnicity with a variable for whether or not an individual identified with the most frequent, White ethnic group. We found no significant association between this variable and the number of other‐author papers (*p* = .3; *n* = 131).

#### 3.1.2 Number of applications made before obtaining a postdoc

We found a significant association between the total number of papers and the number of postdoc applications, such that individuals reporting a greater combined number of first‐ and other‐author papers make fewer applications, before obtaining a postdoc, than those with fewer publications (estimate = −0.07; *p* = .02; Table 2).

#### 3.1.3 Types of contract

As expected in the fields of ecology, evolutionary biology and behavior, those who had permanent contracts were more likely to report having teaching and research contracts than research‐only contracts (estimate = 5.17; *p* < .001). Individuals from a lower socioeconomic background were also significantly more likely to report having teaching and research contracts than research‐only contracts, after accounting for job permanency (estimate = 1.61; *p* = .03; Table 2).

There was a positive association between the total number of publications reported and the probability of securing a permanent academic position (estimate = 1.19; *p* < .01). There was also evidence to suggest a negative association between age and the probability of securing a permanent position, such that individuals aged younger than 34 were less likely to report securing a permanent position (estimate = −1.90; *p* < .001; Table 2).

#### 3.1.4 Number of grant applications made

There were no significant associations between the characteristics of interest and the reported number of grants applied for (all *p* > .05). As expected, there was a significant temporal effect, with those who submitted their PhD earlier reporting having made significantly more grant applications than those who handed in their PhDs later (e.g., years 2007–2009 vs. years 2014–2015; estimate = −1.07; *p* < .001; Table 2).

### 3.2 What barriers do ECRs report as having been detrimental to their career?

LGBT+ individuals were significantly more likely to report facing a barrier than heterosexuals (estimate = 3.91; *p* < .01). Females were significantly more likely to report facing a barrier than males (estimate = 2.30; *p* < .001). Finally, individuals from a lower socioeconomic background were significantly more likely to report facing a barrier than those from a higher socioeconomic background (estimate = 1.93; *p* < .01; Table 2).

Of the 71 free text responses received to the question “What do you feel was the most important [barrier]?”, the most frequently used words were related to sex; “woman/women” (*n* = 17), “family” (*n* = 10), “gender” (*n* = 10), “female,” and “male” (both, *n* = 7). The use of these words was associated with the sex of the respondent (chi‐squared test; *p* = .01), with 60% of females using one of these words in their response, compared to just 15% of males.

### 3.3 Overcoming stated barriers

There were 55 free text responses to the question “If you were able to overcome the barrier stated in the previous question, how?” Of these, almost a third (*n* = 18) reported that they had not overcome their barriers and/or had left an institution, or academia all together, because of them. A number of words appeared at frequencies of 3–5, which can be divided into main categories “people” and “opportunities.” The “people” category included phrases related to support, avoiding judgmental people, meeting people networking, associating with “high quality” groups and senior allies, and mentoring. In terms of “opportunities,” suggestions that appeared several times were the importance of taking up opportunities, proving one's self and skills, working hard, applying for many grants and positions, moving between institutions and asking for opportunities, both in negotiations, but also more generally. Other comments made with respect to opportunities, albeit less frequently, included the importance of perseverance, ensuring CVs are maintained well, participating in departmental activities, and seeking out paid work experience.

## 4 DISCUSSION

### 4.1 Our findings

We found that ethnic minority groups reported fewer other‐author publications upon finishing their PhD. Coming from an ethnic minority has been reported elsewhere as having a negative impact on career progression (e.g., Fang et al., 2000; Palepu et al., 1998), with ethnic minorities more likely to experience institutional and cultural barriers to career progression (Alexander & Arday, 2015; Rollock, 2019). Ethnic minority academics are less likely to have role models; more likely to suffer from imposter syndrome; more likely to lack a sense of “belonging” in academia; and less likely to be promoted (Abelson et al., 2018; Bhatt, 2013; Kameny et al., 2014; Rollock, 2019). However, we suggest that larger sample sizes are necessary to confirm this association, while accounting for the region where PhD was awarded. In addition, our analyses found an indication that socioeconomic background was also a predictor of numbers of papers (relative importance of predictor = 0.43). Furthermore, people from a lower socioeconomic background were more likely to report being in teaching and research positions as opposed to research‐only positions. Research‐only positions are perceived as more prestigious by some institutions. In contrast, teaching‐only contracts (which we were unable to include in our analyses due to a small sample size) can be associated with decreased job security and satisfaction, as many teaching positions at UK institutions tend to be fixed contract and do not have routes for promotion.

Socioeconomic background as an important determinant of career progression has been acknowledged elsewhere (e.g., National Academy of Sciences, National Academy of Engineering, & Institute of Medicine, 2011), but the importance of financial support has received little widespread discussion in the wider STEM academic community, possibly because it is something of a sensitive topic. Given the precarious nature of STEM careers—often involving short‐term contracts, and lengthy periods of unemployment and/or frequent geographic relocations between contracts—it is logical that familial wealth could prove a key determinant of whether an individual is able to progress to the next stage of their career. In addition, the culture of academia is one historically more associated with the upper classes, and individuals from a lower socioeconomic background are, perhaps, more likely to struggle with a lack of relatable role models, difficulty “fitting in,” and imposter syndrome (Gardner & Holley, 2011; Lott, 2002).

Financial barriers may be particularly relevant to ecology, evolution, behavioral ecology, and related disciplines, due to research in these fields often relying on field work. Experience with fieldwork can be key to career development; however, gaining this experience often requires undertaking voluntary internships, which may only be accessible to those from more privileged backgrounds (Fournier & Bond, 2015). In addition, undertaking unpaid work early on in a career has been shown to be related to lower persistence in academia (Fournier et al., 2019). The importance of socioeconomic background is worrying given increasing inequality and economic instability worldwide.

Although we found no direct relationship between the characteristics of interest and job applications, we did find that reportedly having fewer publications on finishing a PhD translated into having to apply for more positions in order to secure a postdoctoral job. Similarly, reporting more publications translated into an increased likelihood of securing a permanent position. It is therefore likely that the effect of these characteristics on publication record could impact indirectly on future job applications and create a knock‐on effect at a later career stage.

We were interested in studying multiple characteristics, including interactions between them, to explore whether barriers to career progression are compounded for individuals who identify with more than one characteristic. As an example, ethnic minority women were the least represented group in UK academia in 2016–2017, with only 25 black female professors out of 19,000 at that time (Abelson et al., 2018), and reported considerable barriers to career progression (Rollock, 2019). Quantitative analysis of the experiences of such underrepresented groups is often difficult due to small sample sizes. We came up against the same problem here with, for example, just eight disabled females having completed our survey. It is perhaps not surprising then that we did not find any evidence in our models for significant interactions between sex and the other characteristics of interest. Once again, we suggest that larger sample sizes are necessary to say anything conclusive about the importance of these interactions, and intersectionality more generally, for ECR career progression.

We found that multiple characteristics were important for predicting if an individual reported a barrier. Respondents that were female, LGBT+, or came from a lower socioeconomic background were the most likely to report having faced a barrier. Furthermore, barriers related to sex were most frequently cited, particularly by female respondents.

### 4.2 A toolkit for overcoming barriers

Worryingly, almost half of the responses concerning “overcoming barriers” were from respondents who stated they had left academia due to a barrier they had not been able to move past. However, other respondents made suggestions for overcoming barriers which we divided into two main categories: “people” and “opportunities.” With regard to “people,” several respondents mentioned mentoring, and indeed, there is a wealth of literature that suggests that effective mentoring can be beneficial at all stages of a career (e.g., Eby et al., 2008; Hunt et al., 2018; van der Weijden et al., 2015). Seeking senior allies and networking were also mentioned. Evidence suggests that the establishment of professional networks both inside and outside of the institution can be beneficial to career success (e.g., Hadani et al., 2012; Parker & Welch, 2013), and diverse networks have been shown to be particularly advantageous (Spurk et al., 2015). Physical attendance at conferences is an obvious route to networking; however, increasingly digital methods of building networks are also available for women (e.g., SciSisters for female academics based in Scotland, http://www.chemicalimbalance.ed.ac.uk/scisister/ and 500 Women Scientists, https://500womenscientists.org/ which is worldwide), LGBT+ academics (e.g., The British Ecological Society LGBT+ Network https://www.britishecologicalsociety.org/membership‐community/diversity/), and ethnic minority academics (e.g., the Twitter forum Minorities in STEM, https://twitter.com/minoritystem?lang=en). More opportunities for such digital networking are needed worldwide.

Regarding the “opportunities” category, many responses highlighted the importance of perseverance, working hard and always to a high standard, publishing as frequently as possible, applying to as many positions as possible, and ensuring CVs are maintained well. Proactive participation in departmental activities was deemed important by one respondent, while another suggested seeking out paid internships to gain work experience. These constructive suggestions are common themes in advice for overcoming bias in the workplace, although it is important to recognize the role of institutions in ensuring such opportunities are made accessible to all early career STEM academics; institutional cultural change is needed to ensure that minority groups do not have to work harder than nonminority groups to succeed or prove themselves.

### 4.3 We have not won the battle for gender equality

It is worth highlighting that while sex featured strongly in the self‐reported perception of barriers, it was less significant in predicting (objective) measures of career progression. It is possible that respondents felt more confident discussing gender in the free text comments as there is now a widespread narrative with regard to women in science. We also note that we received more responses to our survey from females ECRs than male ECRs. In contrast, the other characteristics, such as coming from a lower socioeconomic background or being from an ethnic minority, have received less attention, and so potentially people view these as more sensitive and are less comfortable expressing their opinions.

We find it worrying that gender is still deemed a significant obstacle to career progression, suggesting that UK initiatives such as Athena SWAN have much work still to do.

## 5 CONCLUSIONS AND MOVING FORWARDS

Our models suggest a role for all the characteristics of interest in ECR career progression, suggesting that ultimately there is a significant pool of the workforce who are struggling to access, retain, and succeed in an academic career. As reported elsewhere, the challenges faced by individuals from minority groups are not only leading to a loss of diversity in the workplace, but also to the loss of talented individuals who could and should be meaningfully contributing to higher education (Intemann, 2009). In addition, there is an important moral argument to be made for ensuring opportunity in academia is accessible by all.

We should be concerned that the picture may be even bleaker than it seems; our sampling method, that is, distributing our survey via academic channels, was unlikely to capture responses from many individuals who have already left academia as a result of the barriers they faced. In addition, the sample size for some groups, particularly those relating to disability and sexual orientation, were extremely small and this limited our ability to reliably analyze questions relating to these groups. Low representation of some protected groups in academia may be one reason why so much of the research carried out relies to some extent on qualitative rather than quantitative analysis. Indeed, we could not include some of the subgroups with lowest representation in our quantitative analyses (due to problems with model convergence). It is concerning that studies like ours are likely to exclude the most marginalized groups from analysis for practical research reasons. In doing so, we overlook the groups arguably in most need of consideration.

More broadly, we recognize that our broadcast method of using social media and the EvolDir mailing list had its limitations; while the email list for EvolDir comprises over 10,000 users, our response rate of 188 represents a very low proportion of the total number. We also appreciate that our sample of responses may have been biased in terms of who engaged with the original emails about the survey, who actually responded, and the answers that they gave. For example, individuals that have had negative experiences may have been more likely to respond. That said, we did have approximately equal numbers of respondents reporting having faced a barrier or not (see above).

Clearly, the ecology and evolution community, and the STEM academic community more widely, has work to do. Community initiatives are making strides in breaking the barriers that face a substantial part of the academic population, but further support is needed at all levels. Nationally, we need to ensure that access to education and retention in the academic pipeline is inclusive to all. Individually, institutions have an important role in ensuring accessibility and inclusivity for student and staff hiring, retention, and management. We hope this study stimulates open discussion and further research into this area.

## CONFLICT OF INTEREST

None declared.

## AUTHOR CONTRIBUTIONS

**Klara M. Wanelik:** Conceptualization (equal); Data curation (equal); Formal analysis (equal); Investigation (equal); Methodology (equal); Writing‐original draft (equal); Writing‐review & editing (equal). **Joanne S. Griffin:** Conceptualization (equal); Data curation (equal); Formal analysis (equal); Investigation (equal); Methodology (equal); Writing‐original draft (equal); Writing‐review & editing (equal). **Megan L. Head:** Investigation (equal); Methodology (equal); Writing‐original draft (equal); Writing‐review & editing (equal). **Fiona C. Ingleby:** Formal analysis (equal); Investigation (equal); Methodology (equal); Writing‐review & editing (equal). **Zenobia Lewis:** Conceptualization (equal); Data curation (equal); Funding acquisition (lead); Investigation (equal); Methodology (equal); Project administration (lead); Supervision (lead); Writing‐original draft (equal); Writing‐review & editing (equal).

## Supporting information

Supplementary Material

## Data Availability

Under the terms of University of Liverpool ethical review application number 2229, an aggregated data set is available on Dryad, accession number https://doi.org/10.5061/dryad.1zcrjdfpn.
