# Supplementary material for: Breaking barriers? Ethnicity and socioeconomic background impact on early career progression in the fields of ecology and evolution
Source: Ecol Evol. 2020 Jun 8;10(14):6870–80. doi: 10.1002/ece3.6423 (PMC7391347; doi:10.1002/ece3.6423)

**Supplementary information**

**Table S1** A breakdown of the 188 respondents to our survey in relation to the characteristics of interest: ethnicity, current age, sexual orientation, sex, disability and socioeconomic background. Not all respondents answered all questions regarding their characteristics. This is reflected in some of the figures.

| **Characteristic** | ***N*** |
| --- | --- |
| **Ethnic group**  Black, Asian and minority ethnic (BAME)  Latino Hispanic  White  Other | 14  15  154  5 |
| **Current age**  18-24  25-29  30-34  35-39  40-44  45-49  50+ | 12  35  77  38  21  4  1 |
| **Sexual orientation**  Straight  LGBT+  Prefer not to answer | 155  24  9 |
| **Sex**  Females  Male  Other | 123  63  2 |
| **Disability**  Yes  No  Prefer not to answer | 12  175  1 |
| **Socioeconomic (SEC) background**  Yes - from a lower SEC  No - not from a lower SEC  Prefer not to answer | 46  139  3 |

**Table S2** Table detailing the global models for each of the questions

| **Response**  **variable** | ***n*** | **GLM Error family** | **Link function** | **Global model** |
| --- | --- | --- | --- | --- |
| ***Publication record*** | |  |  |  |
| No. first author papers | 144 | Negative  Binomial | Log | Sex, Sexual orientation, Ethnic group, Socioeconomic background, Disability, Age PhD, Year PhD, Sex x Sexual orientation, Sex x Ethnic group, Sex x Disability |
| No. other author papers | 144 | Negative  binomial | Log | Sex, Sexual orientation, Ethnic group, Socioeconomic background, Disability, Age PhD, Year PhD, Sex x Sexual orientation, Sex x Disability |
|  | |  |  |  |
| No. applications made before obtaining a postdoc | 126 | Negative  binomial | Log | Sex, Sexual orientation, Ethnic group, Socioeconomic background, Disability, Age PhD, Year PhD, Total publications PhD, Sex x Sexual orientation, Sex x Ethnic group, Sex x Age PhD, Sex x Total publications PhD |
| ***Types of contract*** | |  |  |  |
| Research vs. Teaching & research | 111 | Binomial | Logit | Sex, Sexual orientation, Ethnic group, Socioeconomic background, Disability, Year PhD, Total publications PhD, Total postdocs, Permanent or not, Sex x Sexual orientation, Sex x Ethnic group, Sex x Disability, Sex x Total publications PhD |
| Permanent or temporary contract | 139 | Binomial | Cloglog | Sex, Age current, Sexual orientation, Ethnic group, Socioeconomic background, Disability, Total postdocs, Total publications PhD, Year PhD, Sex x Sexual orientation, Sex x Ethnic group, Sex x Disability, Sex x Total postdocs, Sex x Total publications PhD |
|  | |  |  |  |
| No. grant applications made | 122 | Negative  binomial | Log | Sex, Sexual orientation, Ethnic group, Socioeconomic background, Disability, Year PhD, Total publications PhD, Total postdocs, Sex x Sexual orientation, Sex x Ethnic group, Sex x Disability, Sex x Total publications PhD |
| ***Reported barriers*** | |  |  |  |
| Reported barrier or not | 133 | Binomial | Logit | Sex, Sexual orientation, Ethnic group, Socioeconomic background, Disability, Year PhD |

**Table S3:** Table detailing the top models for each of the questions

| **Response** | **Top model** | **∆AIC** | **Akaike weight** |
| --- | --- | --- | --- |
| ***Publication record*** |  |  |  |
| No. first author papers | (Null)  Ethnic group  Socioeconomic background  Disability, Socioeconomic background  Disability  Ethnic group, Socioeconomic background | 0.00  0.39  0.41  1.24  1.29  1.68 | 0.24  0.20  0.20  0.13  0.13  0.10 |
| No. other author papers | Ethnic group  Ethnic group, Sex  Disability, Ethnic group | 0.00  1.08  1.37 | 0.48  0.28  0.24 |
|  | |  |  |
| No. applications made before obtaining a postdoc | Total publications PhD  Sex, Total publications PhD  Sex, Socioeconomic background, Total publications PhD  Age PhD, Sex, Total publications PhD, Age PhD × Sex  Disability, Total publications PhD  Socioeconomic background, Total publications PhD  Disability, Sex, Total publications PhD  Sex, Total publications PhD, Sex × Total publications PhD | 0.00  0.20  1.30  1.37  1.59  1.65  1.75  1.81 | 0.22  0.19  0.11  0.11  0.10  0.09  0.09  0.09 |
| ***Types of contract*** |  |  |  |
| Research vs. teaching & research | Disability, Sexual orientation, Permanent or not, Sex, Socioeconomic background, Disability × Sex  Disability, Permanent or not, Sex, Socioeconomic background, Disability × Sex  Disability, Permanent or not, Total postdocs, Sex, Socioeconomic background, Disability × Sex  Permanent or not, Disability × Sex  Disability, Sexual orientation, Total publications PhD, Permanent or not, Sex, Socioeconomic background, Disability × Sex | 0.00  0.43  1.04  1.22  1.47  1.71 | 0.26  0.21  0.15  0.14  0.12  0.11 |
| Permanent or temporary contract | Age current, Total postdocs, Total publications PhD  Age current, Total postdocs, Sex, Total publications PhD, Total postdocs × Sex  Age current, Sexual orientation, Total postdocs, Total publications PhD  Age current, Sex, Total postdocs, Total publications PhD  Age current, Disability, Total postdocs, Total publications PhD | 0.00  0.56  1.65  1.69  1.71 | 0.33  0.25  0.14  0.14  0.14 |
|  |  |  |  |
| No. grant applications made | Socioeconomic background, Year PhD  Year PhD  Sex, Year PhD  Sex, Socioeconomic background, Year PhD  Sexual orientation, Year PhD  Sexual orientation, Socioeconomic background, Year PhD  Disability, Socioeconomic background, Year PhD | 0.00  0.42  0.83  1.26  1.62  1.62  1.92 | 0.23  0.19  0.15  0.12  0.10  0.10  0.09 |
| ***Reported barriers*** |  |  |  |
| Reported barrier or not | Sexual orientation, Sex, Socioeconomic background, Year PhD  Sexual orientation, Sex, Socioeconomic background  Disability, Sexual orientation, Sex, Socioeconomic background, Year PhD | 0.00  1.90  1.95 | 0.57  0.22  0.21 |

**Fig S1** Barplot showing, for each geographical region, the proportion of respondents from each ethnic group.


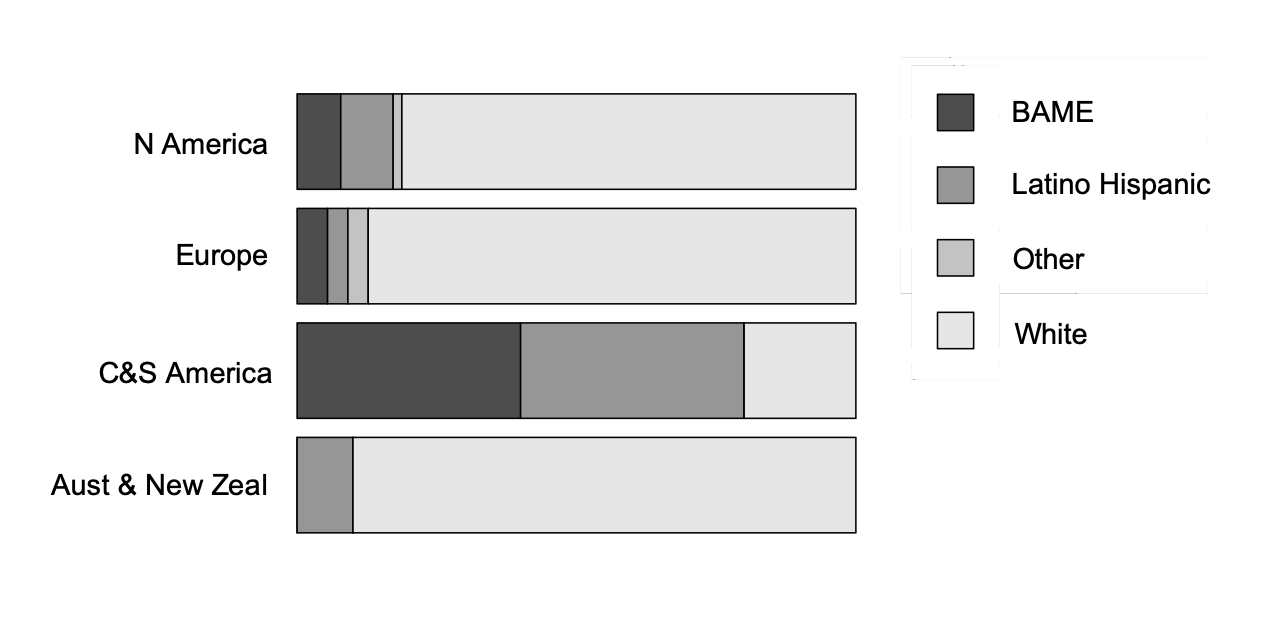

Supplement: Supplementary file 1 — Supplementary Material [file ECE3-10-6870-s001.docx]
